# Planning and executing orthopedic journal clubs

**DOI:** 10.4103/0019-5413.30525

**Published:** 2007

**Authors:** Jaydeep K Moro, Mohit Bhandari

**Affiliations:** Division of Orthopedic Surgery, McMaster University, Hamilton, Ontario, Canada

The earliest reference to a journal club is possibly in the biography of Sir James Paget during the period of 1835-1854.^1^ It was noted that a group of students met in a room over a baker's shop near St. Bartholomew's Hospital to read journals or play cards. However, Sir William Osler was credited to have conducted the first journal club on record in 1875 in North America.^1^,^2^ Its purpose was “for the purchase and distribution of periodicals to which he (Osler) could ill afford to subscribe as an individual”.^1^ Osler then transferred to Johns Hopkins University Medical School in 1889 where he established the Book and Journal Club. There, over dinner, the members recommended new books for the library and reviewed the latest in medical literature.^3^ Journal clubs have now become ubiquitous in graduate medical education training programs, including orthopedic surgery.^4^ Greene surveyed 149 North American orthopedic surgery training programs and found that 99% included a regularly scheduled journal club.^4^ They initially served to help practitioners keep up to date with the latest published literature.^1^,^5^–^7^ More recently, however, they have been used to teach critical appraisal skills, research design, medical statistics, clinical decision theory, clinical epidemiology and encourage “evidence-based” medical practices.^8^

The purpose of this article is to provide organizers with tools to plan and execute successful journal clubs in orthopedic surgery training programs. Guyatt noted that not all clinicians need to appraise evidence from scratch, but all need some skills.^9^ Thus, each journal club needs to respond to the needs of its participants and thus no single ideal format exists.

## PURPOSE AND GOALS OF JOURNAL CLUBS

Originally, journal clubs were established for keeping abreast with the current literature. This has become an unrealistic goal with the explosion of orthopedic journals. Current goals include promoting evidence-based orthopedics and learning general critical appraisal skills for residents. Evidence-based medicine (EBM) is becoming an accepted educational paradigm in medical education.^10^,^11^ EBM relies on the conscientious, explicit and judicious use of current best evidence in making decisions about the care of individual patients.^12^ There are four key elements of EBM: question setting, literature searching, selecting relevant papers and critical appraisal. Historically, internal medicine training programs in Canada and the United States have included the use of EBM practices. Recently, surgical training programs have started to include these principles into their curricula. Evidence-based surgery is often poorly incorporated into orthopedic residency clinical training. Journal clubs can be an ideal vehicle for evidence-based orthopedic surgery education.

Several articles have studied the importance of teaching critical appraisal skills to surgical residents.^4^,^13^–^15^ This goal was ranked the most important in Greene's survey of 149 program directors of North American orthopedic training programs.^4^ Other important goals identified in his study were instilling the habit of reading scientific journals (20% of programs) and facilitating residents learning about current research (9% of programs).

Journal clubs in general surgery were surveyed with questionnaires filled out by program directors in American institutions.^14^ A response rate of 80% from 278 programs identified that 64% of directors thought their journal club was important or very important to the educational mission of their training program. Other top purposes included learning literature review skills (87.5% of directors), providing training in research education (52.9%) and research design (52.4%).

Clinical knowledge transmission was found to be a more important goal in journal clubs in orthopedic fellowship programs over residency programs. This was found in a survey of 57 hand surgery fellowship directors, where the current literature was reviewed for the fellows and faculty as a primary goal.^15^ These fellowship directors felt this journal club was very important for fellowship training.

Secondary goals of journal clubs for residents include increasing reading comprehension, fostering resident-faculty relations and learning orthopedics in an alternative manner.^4^,^14^

In American internal medicine residency programs, national accreditation requirements mandate that residents participate in journal clubs regarding the critical assessment of the literature, clinical epidemiology and medical statistics. This may become mandatory in orthopedic surgery training programs in the future. In addition, faculty members can fulfill their continuing education requirements if mandated by their national colleges. National qualifying examinations in some countries for some specialties may include critical appraisal of a paper, as seen in psychiatry.^16^

The target audience must be identified, including their needs and interests. This will influence the format of the journal club and papers chosen. Emphasis may be placed on keeping up to date with recent advances in orthopedics versus the educational value of critical appraisal of papers. These goals are not mutually exclusive, but each requires significant time and organizational commitments.

## PREVALENCE AND CHARACTERISTICS OF ORTHOPEDIC JOURNAL CLUBS

There are only two known published papers regarding orthopedic journal clubs. In 2000, Greene was the first to survey 161 North American orthopedic training program chairmen.^4^ One hundred and forty-nine programs (93%) responded. Regularly scheduled journal clubs were part of the educational program in 99% of the orthopedic residency training programs. Seventy-eight per cent met once per month and 9% met twice per month. Papers from more than one scientific journal were reviewed in 82% of clubs. Half of the meetings (50%) restricted discussion to scientific studies only. A minority of clubs discussed case reports or review articles. The average number of assigned papers to each resident was 2.6 (range: 1-8). A designated faculty member (38%) or the chairman of the orthopedic program (17%) moderated the discussion, whereas 32% were moderated by rotating faculty and 13% by chief residents. Half of the meetings were held at the departmental office, 31% met at the home of a faculty member and 18% met at a restaurant. The meetings were held in the evening (68%) or at the start of the work day (29%). All journal clubs met for a period of one to two hours. Attendance by the residents at the club was rated as 80% to 100% by 59% of programs, 60% to 80% by 36% of programs and 40% to 60% by 4% of programs. A full meal was provided at 50% of the meetings or a light snack provided at 37%, mostly funded by departmental funds. The top three goals of these clubs were: teaching residents how to evaluate scientific articles, instilling a habit of reading scientific journals and facilitating residents learning about current research.

Dirschl, Tornetta and Bhandari published a comprehensive review of orthopedic journal clubs in 2003.^17^ These authors discussed methods to design, conduct and evaluate these educational meetings with current evidence. The following discussion will address these issues.

## PLANNING AND ORGANIZATIONAL ISSUES

Not all journal clubs are successful despite their presence in the majority of the orthopedic training programs. Sidorov^8^ defined successful journal clubs as those that meet educational objectives and as those that continually promote and maintain resident interest. Jones *et al* suggested that all departments with a journal club should regularly revise their selection of journals in order to increase the value of this important educational process.^18^

## LEADERSHIP

The responsibility of running the journal club by a designated individual or group of individuals is important. Effective journal clubs are correlated with having a designated leader.^5^ An interest in medical education and a belief that the journal club is important are essential attributes of the leader and facilitators. This person or persons would be responsible for the organization, execution and evaluation of the club. In addition, having residents actively involved in planning and operating the club has been found to be important.^19^,^20^ Having a skilled moderator, whether resident or faculty member, is important to the value and attendance of the club.^17^

Part of the success of any journal club is the organization within which it is run. If the orthopedic division or department supports this activity, then it is more likely to be successful.

## LEARNING OBJECTIVES

Although many studies state educational goals for the journal club, many programs do not have formal written learning objectives. This was demonstrated in a survey of emergency medicine program directors, where 42% of programs had not established objectives.^19^ These objectives are important to formalize as they articulate and communicate the goals of the club. Parameters of the meeting can be defined regarding the content, number of papers to be reviewed, which journals' papers should be obtained from, the format and setting. The effectiveness of the club can then be measured based on these objectives.^17^

## ENVIRONMENT

Maintaining boundaries is important to the success of the club. Thus trainees should be relieved from clinical duties, except emergency patient care responsibilities, especially with support from those faculty members not attending the journal club. This aids the session to start and stop on time. Papers should be distributed well in advance to attendees and the room should be comfortable. By maintaining clear boundaries, group theory suggests that the environment allows a sense of security and thus allows creative thinking.^16^

Audience participation is crucial to the success of journal clubs, unlike traditional teaching conferences. It has been shown that interventions involving only didactic sessions do not change physician behavior.^21^ The educational value of the meeting is optimized by the interactions of the attendees and the dynamic exchange of opinions and ideas.^17^

Mandatory attendance has been found to be important^8^ with respect to high attendance rates and longevity of journal clubs. Continuity is promoted by having a regular group of attendees. On the other hand, trainees may feel attendance is imposed on them rather than viewing the club as an educational opportunity. This may be addressed by allowing residents to set their own rules such as journal club presenter opportunities and participating in choosing papers to discuss.

The venue should suit the group. It should facilitate relaxation and conversation. Promoting eye contact and encouraging active participation are facilitated by seating individuals in a circle or a horseshoe.^16^,^17^ Options for meeting locations include departmental offices or conference rooms, faculty homes or restaurants.^4^ Restaurants or pubs may be distracting if there is significant ambient noise or music drowning out the discussion of papers.

Optimal discussion groups are composed of 10 to 12 members.^17^ Thus in large journal clubs, it may be necessary to divide attendees into these smaller groups to facilitate participation and discussion amongst all members.

## MODERATORS

Faculty support with independent running of the journal club (by a senior trainee) was shown to lead to high attendance rates.^8^,^20^ Having a trainee as a club leader may make the fellow trainees feel less intimidated in asking questions. Senior trainees, rather than the faculty, may be more attuned to the needs of their junior colleagues.

## FREQUENCY

Monthly journal clubs are the norm for the majority of postgraduate training programs.^4^,^22^ More frequent meetings may be difficult, especially if residents are expected to review numerous papers for each club or if a detailed critical appraisal is required.

## TIME

Residents and faculty are busy attending to clinical duties during daytime working hours in the majority of orthopaedic training programs. Thus having journal clubs early in the morning before the work day or in the evenings is more convenient for all involved.^17^ In contrast, midday meetings may be more convenient in nonsurgical training programs.

## FOOD

The regular provision of food has been associated with successful journal clubs.^4^,^22^ In orthopedics, 87% of meetings provide some sort of food.^4^ Longevity and high attendance rates are correlated with the provision of food.^8^ Funding journal club meals may be provided by individual faculty or by divisional or departmental support. There have been recent strict North American guidelines on industry funding of medical educational events. This same issue has been discussed in the United Kingdom.^39^

## NUMBER OF PAPERS TO REVIEW

There is significant variability in the number of papers to review in journal clubs.^15^,^23^,^24^ A range of three to 10 papers seems to be a reasonable trade-off between having too many articles (limiting valuable discussion) and too few articles (limiting the breadth of information).^17^ The number of papers and depth of review should be individualized by each training program.

## LINKING JOURNAL CLUB TO THE CURRICULUM

As mentioned earlier, journal clubs can be a venue to teach critical appraisal skills to residents, either as formal sessions or through discussion of the chosen papers. Supplemental formal lectures on this topic could also be provided in the core curriculum of training programs. This has been an important stated goal of orthopedic program directors.^4^ Residents can also be evaluated in journal club sessions regarding national college training requirements, such as the core competencies from The Royal College of Physicians and Surgeons of Canada Canmeds program or The American Council for Graduate Medical Education. If this is an important goal for program directors, establishing formal objectives and evaluation of the journal club would aid in an external review of the training program by their respective national college.

Many authors have studied the educational effects of journal clubs in residency training programs. In one study, residents whose meetings focused on epidemiology and biostatistics thought they read with more attention to the study design and methodology, in self-assessment, than the control group of residents in a traditional journal club.^20^ In this randomized study, there were no significant differences between the two groups of residents in objective testing of knowledge of epidemiology, biostatistics, nor critical evaluation.

In a similar study of emergency medicine residents with a prospective, case-controlled design, the study group (journal club with emphasis on epidemiology principles) and control group (traditional journal club) of residents improved their performance over the one-year period.^25^ No difference was shown between groups with objective pre-testing and post-testing in gaining knowledge in clinical epidemiology principles. This similar result was found in another study of two pediatric training programs, whose only difference was that one program had two introductory lectures on epidemiology principles.^26^

## IMPACTS OF JOURNAL CLUB ON READING HABITS, KNOWLEDGE AND SKILLS

Parkes *et al* revealed a small evidence base that journal clubs probably do improve knowledge in biostatistics and clinical epidemiology in a Cochrane review.^27^ Conflicting evidence exists on whether they enhance critical appraisal skills. Self-assessment shows more improvement than objective measurement of skills.^22^ The impact of journal clubs on patient outcomes has not yet been investigated in a well-designed trial, as there is difficulty in measuring this process.

## STRUCTURED REVIEW INSTRUMENTS

Prepared checklists exist to review papers in a structured format. These can be found in reading guides such as the Users' Guide to Medical and Surgical Literature series of articles in the Journal of Bone and Joint Surgery,^28^ textbooks^29^ and the McMaster reading guides (Department of Clinical Epidemiology and Biostatistics, McMaster University, 1981). The advantage is that the reader can distinguish between proper and poor methodology in a more consistent manner.

Guidelines and dataforms for critical appraisal of published articles may be downloaded from the Oxford University evidence-based medicine website at http://www.cebm.net/downloads.asp.

Alguire *et al*^22^ published a packaged critical appraisal curriculum that included reading guides and other teaching materials.

The use of a reading checklist was evaluated in an emergency medicine training program.^25^ Resident use of the structured review instrument was associated with higher overall satisfaction with the journal club format and a perceived improvement in clinical education. Improvement in critical appraisal skills was not tested. The residents did not perceive an increased workload, nor did attendance rates decrease.

Thus the use of a structured review instrument is an effective adjunct to the journal club for teaching and applying critical appraisal. This tool may be used for resident evaluation of knowledge acquisition and the impact of the meeting on the overall educational training program.

## TYPES OF JOURNAL CLUBS

Many types of journal clubs exist. No one type is superior to the others. This allows organizers to tailor the club to their educational objectives. An individual residency training program may choose to adopt more than one type, as each one may not address all goals and objectives desired. Innovative types of journal clubs used elsewhere may generate ideas for local use.

## EVIDENCE-BASED JOURNAL CLUBS

EBM relies on the conscientious, explicit and judicious use of current best evidence in making decisions about the care of individual patients.^12^ There are four key elements of EBM: question setting, literature searching, selecting relevant papers and critical appraisal. Thus EBM journal clubs are a more extensive undertaking, with critical appraisal being just one key element. Thus a wider spectrum of skills is required.

Question setting can start with a real life clinical scenario. These scenarios may come from the outpatient clinic, emergency room or the operating room.

Literature searching is the next step in this format. If no one is confident in this skill, local librarians may help in finding the relevant papers for a topic. Knowledge of search strategies and filtering techniques are important to select relevant articles. Interestingly, in a study by Coomaraswamy *et al*^30^ of journal clubs in obstetrics and gynecology, 11 of 55 consecutive appraisals missed the article most relevant to the clinical question. Critical appraisal then follows with a structured review instrument. Critical appraisal is the systematic weighing up of evidence. The principles of research methods and statistics are the focus.

There are several possible formats to run the EBM journal club. One session may focus on question setting and identifying the most relevant papers. The next session can appraise them. Secondly, attendees can prepare the first three steps in advance, leaving the club's time for critical appraisal. Lastly, a question could be posed for a debate between two teams. A chairperson could mediate arguments from each side.

An evidence-based journal club has been designed for faculty interested in medical education, which can be applied to almost any clinical topic or specialty area.^8^ Here, unread journals relating to a specific topic area are brought to the conference. The attendees read the titles of the papers out loud in small groups and ask three questions to evaluate if specific papers achieve relevancy criteria: 1. Does the article have an impact on the topic? 2. Does the article focus on a common issue? 3. Will the article change practice? The article is tagged for critical review if the answer is yes to all three questions. It is then assigned to two attendees for review and discussion at the next meeting. Challenging the participants to think critically about which article to read was linked to high satisfaction levels.

In an obstetrics and gynecology program, a very structured 12-month evidence-based curriculum has been designed to cover 24 topics in epidemiology, biostatistics and experimental design.^31^ The journal club met monthly for two hours and two sets of papers were distributed for each session. The first set consisted of literature on topics in epidemiology, biostatistics and experimental design, with two to six papers distributed for each session. The second set of papers consisted of articles for critical review, selected from all medical specialties to illustrate the concepts learned from the first set of papers for each session. It was reported as well received by the residents, although a great deal of preparation was necessary. The residents commented that they would have preferred all of the papers to have come from the obstetrical and gynecological literature instead of from general medicine. The full list of papers can be reviewed in this report.^31^

Most EBM occurs in a journal club format.^11^ Training programs may offer a seminar series, where residents present cases, generate questions and critically appraise the literature. Simpler concepts of EBM may be assigned to junior level residents, such as therapy and diagnosis. More complex topics, such as meta-analysis, decision analysis and economic analysis, may be assigned to senior level residents.

It should be noted that not all residents or faculty are interested in attaining an advanced level of EBM skills.^9^ Thus other sources of papers may include the use of evidence-based summaries written by others, such as the evidence-based orthopedics section of the Journal of Bone and Joint Surgery. Learning a basic subset of skills will help in training the next generation of orthopedic surgeons to be up-to-date practitioners, who are referred to as evidence-based users.

## JOURNAL CLUBS BASED ON CRITICAL APPRAISAL

To reiterate, critical appraisal is the systematic weighing up of evidence. The principles of research methods and statistics are the focus of this format. A structured review instrument is used. Up to five papers are reviewed at each meeting. Classic papers may be reviewed, which are frequently referred to in the literature. Attendees can then assess the validity of the conclusions drawn.

By dividing attendees into small groups of 10 to 12 members, more audience participation may occur.^12^,^17^ Each group is assigned critical appraisal questions after the presenter briefly introduces the paper. The session is completed with a general group discussion of the correct answers. The Pocket Guide to Critical Appraisal^32^ may aid in selecting appropriate questions.

One disadvantage of this format is that attendees, including faculty members, may feel intimidated to participate if they don't feel confident with their critical appraisal skills.

Inviting a statistician to the journal club will aid in understanding statistical issues.^16^ An article from internal medicine^33^ reported such a format. One resident volunteers to select and present one clinical study for the journal club. All residents are provided with the paper two weeks before the meeting. At the same time, the resident and a faculty mentor devise and provide a series of questions to all participants regarding the quality of the research, appropriateness of the data and methods and the validity of the conclusions. At the journal club, discussion focuses on answering the questions rather than the clinical aspects of the paper. A great deal of faculty involvement and facilitation is required to succeed in this format.

## JOURNAL CLUBS BASED ON KNOWLEDGE TRANSMISSION

This may be the most popular form of journal club in most training programs. Despite many formats that exist, the main goal is to provide a brief overview of new developments in a topic area or subspecialty.

A survey of orthopedic hand surgery fellowship programs revealed that as many as 12 papers are discussed in a one-hour conference.^15^ The most important goal of these meetings is to review the current literature in hand surgery. A slight modification of this format is to have each attendee review one paper from a journal or topic area.^17^ Each attendee then presents to the group the key contributions of the paper to the topic or specialty area.

## THE NO PREPARATION JOURNAL CLUB

In this unique format, the paper(s) for discussion is not distributed to the attendees prior to the journal club.^24^ Here, a resident chooses a paper and then discusses it at the meeting with the aid of a faculty moderator. The attendees do not read the paper beforehand. A brief presentation of the research question of the selected study begins the meeting. The participants are then asked to suggest appropriate study designs to address the research question, followed by the author's chosen study method. The attendees are then asked to assume the author's chosen study design and suggest and discuss additional details of study methodology. This process continues until all relevant aspects of the study methodology are discussed. The results and conclusions are then reviewed. It was reported in this article that more positive discussion of methodological issues occurred rather than participants being hypercritical, which occurs when papers are reviewed before the journal club meeting. As well, resident workloads were decreased by not having to prepare in advance of the meeting, attendance rates were higher and residents gained a greater appreciation of the author's design choices.

## INTERNET-BASED JOURNAL CLUBS

An otolaryngology residency program reported on a journal club employing e-mail and the internet to archive current literature reviews in otolaryngology.^34^ Each training year in the residency program (PGY2, PGY3), was assigned a particular journal to review. The residents in each year divide the articles in their assigned journal and each resident prepares written reviews of the article(s), usually one to five papers per month. Each resident then submits their review(s) via e-mail to all residents and faculty in the department. Thus a complete review of the current month's otolaryngology literature is provided. The best four to 10 articles are then selected to discuss at that month's journal club. These monthly reviews are archived on a server, which are accessible from the departmental website to all residents and faculty. It can also be searched by title, date, journal and thread.

## EVALUATION OF THE JOURNAL CLUB

Regardless of the format(s) chosen, periodic restructuring of the club will be necessary to meet new goals, as is done for all educational activities. This will help to maintain enthusiasm and effectiveness. To evaluate the effectiveness on individuals' learning, Morrison *et al*^35^ have developed a list of appropriate questions.

Periodically asking the attendees about their satisfaction with the club is one way to modify the meeting if needed. Anonymous feedback from the residents may alert organizers about strengths and weaknesses and potentials for improvement.^17^ The residents could also be asked to self-assess their clinical reading habits and critical appraisal skills. Objective measures of understanding critical appraisal skills could be performed to document knowledge gain in epidemiologic principles, with a pre-test and post-test format^24^ or by the evaluation of a factitious standardized article.^23^,^25^ These periodic evaluation methods will aid in improving the educational experience of the journal club, regardless of its format.

## CATALOGUING CRITICALLY APPRAISED ARTICLES FROM JOURNAL CLUB

Critically appraised topics (CATs) is a term regarding a document one creates for themselves in response to a clinical question. It summarizes an individual item of evidence one has found and presents the results in an easily readable format. General internal medicine fellows at McMaster University invented CATs as a means to sharpen their critical appraisal skills and improve their abilities as bedside teachers of evidence-based medicine. Several medical training programs maintain internet-based logs of papers that have met predetermined criteria for validity on various topics. Thus a database is created of current evidence for access by residents, fellows and faculty.

## JOURNAL CLUBS AND ADULT LEARNING

Educational research has identified several unique characteristics of adult learners. These principles include: relate the task to personal goals or to the immediate environment, present learning objectives as clinical problems, use problem-solving strategies, vary teaching approaches to suit different learning styles, use active learner participation and provide frequent constructive feedback.^16^ It may be that instruction that incorporates these principles will enhance the learning experience for residents.^22^ Skills and behavior, not only knowledge, are likely to be improved when incorporating principles of adult learning.

## CONCLUSIONS

Journal clubs are integral to graduate medical education, including most orthopedic training programs. This is true today as it was when journal clubs were first started. Successful journal clubs can be defined by many different criteria. Factors associated with high attendance and longevity of the club include mandatory attendance, availability of food, perceived educational value of the program director,^22^ having a committed leader, good faculty participation and attendance and formal instruction in epidemiologic principles.^8^,^36^–^38^

Organizers of journal clubs should clearly state goals of the meetings and choose appropriate formats to achieve these goals. It is desirable to attain support from the departmental chairperson and other respected faculty.^38^ Structured review instruments may increase levels of resident satisfaction with the educational experience and increase their understanding of the material and focus their presentations. By evaluating the journal club and responding to the needs of the participants, journal clubs can continue to evolve and improve to remain as a valuable part of the orthopedic residency training program.

## References

[1] Linzer M (1987). The journal club and medical education: Over one hundred years of unrecorded history. Postgrad Med J.

[2] Cushing H (1926). The life of Sir William Osler.

[3] Sleeman W (1990). The book and journal club of the Medical and Chirurgical Faculty of Maryland. Md Med J.

[4] Green WB (2000). The role of journal clubs in orthopaedic surgery residency programs. Clin Orthop Relat Res.

[5] Heiligman RM, Wollitzer AO (1987). A survey of journal clubs in U.S. family practice residencies. J Med Educ.

[6] Helligman RM (1991). Resident evaluation of a family practice journal club. Fam Med.

[7] Mattingly D (1966). Journal clubs. Postgrad Med.

[8] Sidorov J (1995). How are internal medicine residency journal clubs organized and what makes them successful?. Arch Intern Med.

[9] Guyatt GH, Meade MO, Jaeschke RZ, Cook DJ, Haynes RB (2000). Practitioners of evidence based care. Not all clinicians need to appraise evidence from scratch but all need some skills. BMJ.

[10] Elnicki DM, Halperin AK, Shockcor WT, Aronoff SC (1999). Multidisciplinary evidence-based medicine journal clubs: Curriculum design and participants' reactions. Am J Med Sci.

[11] Korenstein D, Dunn A, McGinn T (2002). Mixing it up: Integrating evidence-based medicine and patient care. Acad Med.

[12] Sackett DL, Richardson WS, Rosenberg W (1997). Evidence-based medicine.

[13] Cheatham ML (2000). A structured curriculum for improved resident education in statistics. Am Surg.

[14] Crank-Patton A, Fisher JB, Toedter LJ (2001). The role of the journal club in surgical residency programs: A survey of APDS program directors. Curr Surg.

[15] Melchior JA, Meals RA (1998). The journal club and its role in hand surgery education. J Hand Surg.

[16] Swift G (2004). How to make journal clubs interesting. Adv Psychiatr Treatment.

[17] Dirschl DR, Tornetta P, Bhandari M (2003). Designing, conducting and evaluating journal clubs in orthopedic surgery. Clin Orthop Relat Res.

[18] Jones SR, Harrison MM, Crawford IW, Ali B, Beattie E, Carley S (2002). Journal clubs in clinical medicine. Emerg Med J.

[19] Jouriles NJ, Cordell WH, Martin DR, Wolfe R, Emerman CL, Avery A (1996). Emergency medicine journal clubs. Acad Emerg Med.

[20] Linzer M, Mercando A, Hupart KH (1986). Role of a medical journal club in residency training. J Med Educ.

[21] O'Brien T, Freemantle N, Oxman AD (2001). Continuous educational meetings and workshops: Effects on professional practice and health are outcomes.

[22] Alguire PC (1998). A review of journal clubs in postgraduate medical education. J Gen Intern Med.

[23] Burnstein JL, Hollander JE, Barlas D (1996). Enhancing the value of journal club: Use of a structured review instrument. Am J Emerg Med.

[24] Hartlaub PP (1999). A new approach to the journal club. Acad Med.

[25] Bazarian JJ, Davis CO, Spillane LL, Blumstein H, Schneider SM (1999). Teaching emergency medicine residents evidence-based critical appraisal skills: A controlled trial. Ann Emerg Med.

[26] Langkamp DL, Pascoe JM, Nelson DB (1992). The effect of a medical journal club on residents' knowledge of clinical epidemiology and biostatistics. Fam Med.

[27] Parkes J, Hyde C, Deeks J (2001). Teaching critical appraisal skills in health care settings.

[28] Bhandari M, Guyatt GH, Swiontkowski MF (2001). User's guide to the orthopaedic literature: how to use an article about a surgical therapy. J Bone Joint Surg.

[29] Guyatt GH, Rennie D (2001). User's guides to the medical literature: A manual for evidence - based clinical practice.

[30] Coomarasamy A, Latthe P, Papaioannou S, Publicover M, Gee H, Khan KS (2001). Critical appraisal in clinical practice: Sometimes irrelevant, occasionally invalid. J Royal Soc Med.

[31] Letterie GS, Morgenstern LS (2000). Teaching critical evaluation on clinical literature in an evidence-based environment. J Reprod Med.

[32] Crombie I (1996). The pocket guide to critical appraisal.

[33] Markert RJ (1989). A research methods and statistics journal club for residents. Acad Med.

[34] Kuppersmith RB, Stewart MG, Ohlms LA, Coker NJ (1997). Use of an internet-based journal club. Otolaryngol Head Neck Surg.

[35] Morrison JM, Sullivan F, Murray E, Jolly B (1999). Evidence-based education: Development of an instrument to critically appraise reports of educational interventions. Med Educ.

[36] Spillane AJ, Crowe PJ (1998). The role of the journal club in surgical training. Aust NZJ Surg.

[37] Valentini RP, Daniels SR (1997). The journal club. Postgrad Med J.

[38] (1981). How to read the clinical journals: I. why to read them and how to start reading them critically. Can Med Assoc J.

[39] Moynihan R (2003). Who pays for the pizza? Redefining the relationships between doctors and drug companies 1: entanglement. BMJ.

